# Knowledge, attitudes and behaviours of a sample of Italian paediatricians towards RSV and its preventive strategies: a cross-sectional study

**DOI:** 10.1186/s13052-024-01593-1

**Published:** 2024-02-29

**Authors:** Giulia Congedo, Gaia Surya Lombardi, Doris Zjalic, Mattia Di Russo, Emanuele La Gatta, Luca Regazzi, Giuseppe Indolfi, Annamaria Staiano, Chiara Cadeddu

**Affiliations:** 1 Department of Life Science and Public Health, Section of Hygiene, Università Cattolica del Sacro Cuore, Roma, Italy; 2 Department Neurofarba, Università degli Studi di Firenze, Firenze, Italy; 3 Meyer Children’s Hospital, IRCCS, Firenze, Italy; 4 Department of Translational Medical Science, Section of Pediatrics, Università degli studi di Napoli “Federico II”, Napoli, Italy

**Keywords:** Respiratory syncytial virus, Infectious diseases, Prevention, Immunization, Child health, Public health

## Abstract

**Background:**

Respiratory Syncytial Virus (RSV) infection mainly affects newborns, infants and young children aged < 2 years. Since an RSV vaccine is in the European Medicines Agency’s waitlist validation, nowadays the prevention only includes passive immunization with monoclonal antibodies (mAb). In the present study we aimed at investigating Italian paediatricians’ knowledge, attitudes and behaviours towards RSV and its prevention.

**Methods:**

From February to May 2023, an anonymous online questionnaire, with answers based on the Likert scale, was administered to a sample of Italian paediatricians’ members of the Italian Society of Paediatrics. Descriptive and inferential statistical analyses were performed using STATA 17.

**Results:**

The paediatricians who answered the questionnaire were 507, mostly women (70.6%), aged 30–45 (33.1%), employed in hospitals in 66.6% of cases. The 10.8% of respondents reported that RSV is transmitted only among children younger than 2 years of age and 80.33% of participants that school-age children are not at risk of developing severe forms of RSV disease. The 25% of participants thought that active immunization is currently available to prevent RSV infection and 35.7% that does not exist passive immunization to prevent RSV for infants and newborns aged < 2 years. The 97.5% of physicians managed bronchiolitis cases and 65.6% of participants did not prescribe the administration of mAb. Higher age, seniority and RSV knowledge score were found to be associated with having a higher mAb knowledge score (*p* < 0.001) and having a higher RSV knowledge was associated with a higher mAb knowledge score (*p* < 0.001). The logistic regression model found that the odds of a positive attitude towards mAB knowledge score increased by over 3 times (OR 3.23, 95% CI [1.41, 7.40], *p* = 0.006) for being female and the odds of a positive attitude towards mAB knowledge score increased by almost 10 times (OR 9.73, 95% CI [3.06, 30.89], *p* < 0.001) for a one-unit increase in RSV knowledge score.

**Conclusions:**

Paediatricians’ limited knowledge or awareness could represent a barrier to the implementation of preventive strategies against RSV infection. Strategies to improve paediatricians’ education on RSV prevention are, therefore, crucial.

**Supplementary Information:**

The online version contains supplementary material available at 10.1186/s13052-024-01593-1.

## Background

Respiratory Syncytial Virus (RSV) is a seasonal virus that causes epidemics in temperate regions of the Northern Hemisphere, including Italy. These epidemics typically last for 5 months from November to March, with peaks in January/February [1]. RSV infects respiratory epithelial cells, leading to inflammation, edema, syncytial formation, and necrosis of the respiratory epithelium [2–4]. Its incubation period ranges from 3 to 7 days, and it has a high transmissibility with a basic reproduction number (R0) of 4.5, surpassing that of seasonal influenza, rhinovirus, and rotavirus [5–8].

RSV can affect people of all ages with varying degrees of severity, but neonates, infants and young children under 2 years old bear the heaviest burden in both inpatient and outpatient settings [9–11]. Almost all children experience RSV infection at least once in their lifetime, often presenting as lower respiratory tract infections (LTRIs). RSV is responsible for more than 6 in 10 (63%) acute respiratory infections in children under 5 years old and over 8 in 10 (> 80%) acute respiratory infections in neonates and infants [12]. In the first year of life, RSV causes 80% of hospitalizations for bronchiolitis and 40% of hospitalizations for pneumonia [13–14].

Although there is no national surveillance for RSV in Italy, hospitals have been reporting cases of bronchiolitis since 2004, accounting for approximately 2% of all newborn hospitalizations, with a slight increase in recent years. Interestingly, the rate of acute bronchiolitis was significantly lower (84–95%) in the 2020–2021 season compared to previous seasons, likely influenced by the SARS-CoV-2 pandemic and public health measures [10–17, 1].

Overall, RSV remains a significant health concern, particularly for youngest children. The latest data have highlighted a strong growth in cases during the 2021–2022 biennium, emphasizing the need for ongoing monitoring and preventive strategies.

Most of the hospitalized children are born healthy and/or at term (90–95%), thus not eligible for current prophylaxis [18–21]. At present, in fact, a form of passive immunization is available as a preventive strategy to cope with RSV [22–23]. On 21 August 2023 FDA approved Abrysvo vaccine [24]. On 23 August 2023, the vaccine has also obtained marketing authorization valid throughout the European Union from EMA [25].

The passive immunization with monoclonal antibodies (mAbs) is currently indicated only for preterm babies and babies with other defined health conditions (5–10% of cases) [26]. These drugs are used up to 35 weeks’ gestation and are recommended by SIN (Italian Society of Neonatology), SIP (Italian Society of Pediatrics) and SIMRI (Italian Society for Infant Respiratory Disease) for preterm births < = 35 weeks’ gestation and for children < 2 years of age with RSV risk conditions [1, 27]. Healthy term and late preterm births (90–95% of cases) are not target for these preventive strategies but could benefit from the use of ready-to-market mAb since they have passed the EMA approval stage. In June 2023, the US Food and Drug Administration Advisory Committee unanimously recommended mAb (Nirsevimab) as first immunization against RSV disease for all infants [28–31]. Nowadays, the National Drug Agency (AIFA) has classified Nirsevimab as a Category C product (a drug not covered by the italian NHS), giving to this medication a restricted medical prescription [32].

Currently, only two studies were performed on RSV in Italy [33–34] focused on gaps in knowledge and lack of risk perception regarding RSV, emphasizing the importance of adequate medical education in this area. Starting from these premises, our aim was to analyse through a survey the knowledge, attitudes, practices and preventive strategies towards RSV among Italian paediatricians.

## Methods

### Study design

We performed a cross-sectional study by means of a survey developed based on sound methodology, submitted to paediatricians through the involvement of the Italian National Society of Paediatrics (SIP) of which the participating paediatricians are members. The aim was to assess knowledge, attitudes and behaviour of a sample of Italian paediatricians towards RSV and its preventive strategies. The survey was online from 21 February to 19 May 2023 in the form of a questionnaire available through the Survey Monkey web platform (“https://www.surveymonkey.com/”). The questionnaire was uploaded by the SIP website technical advisors in the section reserved for members. To access the reserved section, it was necessary to be a SIP member and have the appropriate credentials. The survey was also sent out in a dedicated newsletter to all SIP members. The participants could fill out the questionnaire once to avoid having duplicate answers. The collected data were used in an anonymous and aggregate form, in compliance with the EU General Data Protection Regulation n. 679/2016 (D.gls. n.196/2003 “Codice di Protezione in materia di dati personali” (modified by D.gls. n. 101 del 10.08.2018).

### Sample size

As we could not find any reliable estimate of the number of paediatricians and Paediatric resident, we decided to calculate the minimum sample size using the following formula for unknown (or very large) populations:

$$ Sample\, size= \frac{{z}^{2}*p \left(1-p\right)}{{e}^{2}}$$

where *z* is the z-score corresponding to a desired confidence level, *p* is the expected standard deviation of the outcome in the population, *e* is the margin error of the confidence interval of the outcome in the population.

We chose a desired confidence level of 0.95 (alpha = 0.05) corresponding to a z-score of 1.96, an expected standard deviation of 0.5 (which is usually recognized as a safe value when the actual standard deviation is unknown) and a margin error of 0.05 (i.e., 5%). We then computed a minimum sample size of 385 respondents.

The members who have access to the questionnaire was 11,109, of which 7,717 were paediatricians working in hospitals, 1,393 family paediatricians, 1,550 paediatricians working in university hospitals, 399 paediatricians working autonomously, and 50 paediatricians working in different settings.

### Inclusion criteria

All participants were recruited from the National Society of Paediatrics (SIP) living and working in Italy at the time of the survey. Physicians of all age and gender were recruitable in the study. Enrolment was on a voluntary basis with no financial remuneration.

### Instruments

Participants answered the online anonymous survey made of 30 questions divided into four different sections:

- Socio-demographic and occupational information (gender, age, region of residence, specialty, work setting, no. paediatric patients followed in 2021);
- General knowledge about RSV infection and monoclonal antibodies used against this virus;
- Positions toward immunization strategies;
- Behaviours toward treatment and prevention of RSV infection.

The questionnaire was specifically designed by the Authors for the aim of this work. The validity and reliability of the study were tested by a group of 20 doctors, including 10 public health physicians and 10 paediatricians. Data resulting from the questionnaires were recorded and processed in a database. Categorical data were analysed through descriptive statistical methods using frequencies and percentages (N, %). Continuous data were summarized by means and standard deviations (M, SD).

The Likert scale was also used in the questionnaire in order to probe, among paediatricians, general knowledge about RSV, knowledge with respect to monoclonal antibody immunization and attitudes with respect to their administration. Likert-scale answers regarding RSV knowledge (*n* = 12) and mAb knowledge (*n* = 6) were coded as “5” if “strongly agree” and as “1” if “strongly disagree” when the question/statement was correct, while they were coded as “1” if “strongly agree” and “5” if “strongly disagree” when the statement was incorrect. The answers “agree”, “neutral” and “disagree” were coded as “4”, “3” and “2” or as “2”, “3”, and “4” consistently with the type of question (correct/incorrect). Likert-scale answers regarding paediatricians’ attitude towards mAb use (*n* = 3) were coded as “5” when “strongly agree” and “1” when “strongly disagree”, with “agree”, “neutral” and “disagree” coded as “4”, “3” and “2”, respectively. This allowed to produce a quantitative projection of the data coming from Likert-scale questions and to compute RSV knowledge, mAb knowledge and mAb attitude scores for each participant, by calculating mean and SD of the Likert-type statements related to each dominion.

Individual scores concerning RSV knowledge, mAb knowledge and mAb attitude were divided into three categories: score ≤ 2.5, comprised between 2.5 and 3.5 and > 3.5. The cut-off values 2.5 and 3.5 were chosen because they delimited the unit which had the neutral value 3 as central and which represented the interval of undecided. The > 3.5 category included (but was not limited to) the participants who answered obtaining “4” or “5” for all the Likert-scale questions; the 2.5–3.5 category included (but was not limited to) the participants who obtained “3” to all the Likert-scale questions; the ≤ 2.5 category included (but was not limited to) all the participants who obtained “1” or “2” to all the Likert-scale questions. Chi-2 tests were performed to estimate the significance of differences in RSV knowledge score and mAb knowledge score (categorised as previously explained) associated with differences in gender, job setting, age, professional seniority, geographical area, number of RSV cases handled in the previous season, and mAb knowledge score / RSV knowledge score. Logistic regressions were performed considering the mAb attitude score as dichotomous variable (= 0 if mAb attitude score ≤ 3.5; = 1 if mAb attitude score > 3.5). Gender, age, job setting, geographical region, mean RSV knowledge score and mean mAb knowledge score were included in the logistic regression as independent variables. Odds ratios (Ors) and 95% Confidence intervals (95% CI) were computed to estimate the strength and significance of the associations. Associations with *p*-value < 0.05 were considered statistically significant. Statistical analysis was performed using Stata Statistical Software: Release 17 (College Station, TX: StataCorp LLC).

## Results

### Descriptive analysis

#### Socio-demographic and occupational information

The 507 out of a total of 11,109 participants answered the questionnaire, reaching the minimum sample size of 385. 70.6% were women, 33.1% aged 30–45 years, and 51.3% got specialization for more than 20 years. 81.7% of the respondents had no other specializations. The majority of the paediatricians involved worked in the hospital setting (66.6%). The 24.5% of respondents practiced in professional office and 14.1% carried out private practice. The 55.7% of physicians responded from Northern Italy, 23.6% from Central, 20.7% from Southern and 7.9% from Islands [Table 1].

**Table 1:** Socio-demographic and occupational questionnaire section

VARIABLE	ANSWER OPTIONS	ANSWERS	
Gender	Male Female I prefer not to specify	28.21% 70.61% 1.18%	143 358 6
		**TOTAL**	**507**
Age	< 30 30–45 46–55 56–65 > 65	3.55% 33.14% 16.96% 24.06% 22.29%	18 168 86 122 113
		**TOTAL**	**507**
How long have you been specialized in Pediatrics	I am a resident < 5 years Between 5 and 10 years Between 11 and 20 years > 20 years	7.69% 12.62% 12.43% 15.98% 51.28%	39 64 63 81 260
		**TOTAL**	**507**
Any other specializations	Yes No	18.34% 81.66%	93 414
		**TOTAL**	**507**
Type of specialty	Sport Medicine Oncology Community Medicine e Primary care Allergy and Clinical Immunology Hematology Endocrinology Dietology Gastroenterology Cardiology Pulmonology Infectious Disease Nephrology Rheumatology Neurology Child Neuropsychiatry Clinical Pathology and Clinical Biochemistry Anaesthesiology, Resuscitation and IC Medicine Medical Genetics Preventive Medicine and Public Health Legal Medicine Other (to specify) **Residency schools not represented**: Internal Medicine, Emergency Medicine, Geriatrics, Thermal medicine, Dermatovenereology, Psychiatry, General Surgery, Paediatric Surgery, Plastic, reconstructive and aesthetic Surgery, Obstetrics and Gynecology, Orthopaedics and Traumatology, Urology, Oral and Maxillofacial Surgery, Neurological Surgery, Ophthalmology, Otorhinolaryngology, Cardiac Surgery, Thoracic Surgery, Vascular Surgery, Anatomical pathology, Microbiology and Virology, Radiodiagnosis, Radiotherapy, Nuclear Medicine, Audiology and Phoniatry, Physical Medicine and Rehabilitation, Pharmacology and Clinical Toxicology, Occupational Medicine and Medical Statistics and Biometrics	1.25% 1.25% 1.25% 8.75% 3.75% 8.75% 3.75% 3.75% 1.25% 2.50% 7.50% 1.25% 2.50% 1.25% 5.00% 1.25% 2.50% 3.75% 5.00% 1.25% 43.75%	1 1 1 7 3 7 3 3 1 2 6 1 2 1 4 1 2 3 4 1 35
		**TOTAL**	**89**
Work setting	Hospital facility Policlinic Territorial specialist outpatient clinic Private professional practice (freelance activity) Local health Authority (LHA) Nursing Home (RSA)/Hospice/ Long-term care Home care 118/ Out-of-hospital first aid Research organization Other (to specify)	49.47% 17.06% 2.99% 14.07% 4.90% 0.85% 0.85% 0.64% 0.64% 3.20%	232 80 14 66 23 4 4 3 3 15
		**TOTAL**	**469**
Macroregion in which you work	North-West North-East Centre South Islands	32.20% 23.45% 23.65% 12.80% 7.90%	151 110 111 60 37
		**TOTAL**	**469**
Number of paediatric patients followed in 2021	< 500 500–1000 1000–1500 1500–2000 > 2000	22.81% 38.38% 23.88% 7.04% 7.89%	107 180 112 33 37
		**TOTAL**	**469**

The Table 1 displays the socio-demographic variables, options, and response percentages of the questionnaire respondents

#### RSV general knowledge

Average RSV knowledge score was 4.01 (SD = 0.37). The questions about RSV knowledge which obtained the lowest average score were **Q6** (Apnoea may be the only sign present in infants in the diagnosis of bronchiolitis; mean = 3.71, DS = 0.94), **Q7** (Most RSV hospitalizations occur in children who have pre-existing pathological conditions, such as congenital heart disease and bronchopneumodysplasia; mean = 3.46, SD = 1.07) and **Q8** (Most RSV hospitalizations occur in healthy, preterm and full-term infants born not eligible for current prophylaxis, with rates between 70- 90%; mean = 3.74, SD = 0.88). The question about RSV knowledge which obtained the highest average score were **Q5** (Inability to feed is a criterion for hospital admission for a child affected by RSV; mean = 4.41; DS = 0.65) and **Q9** (All newborns and infants experiencing their first RSV season are at risk of developing RSV infections, such as bronchiolitis, during the period of virus circulation; mean 4.25; DS = 0.60) [Table 2] [Additional file 1].

**Table 2:** RSV knowledge, mAb knowledge and mAb attitude scores

Variable	Mean	SD	Classes				
			0 (≤ 2.5)		1 (2.5–3.5)	2 (> 3.5)	Total
Average RSV knowledge score	4.01	0.37	0.00	9.84		90.16	100.00
Average mAb knowledge score	3.70	0.45	0.25	37.38		62.38	100.00
Average attitude towards mAb score	4.20	0.62	0.51	10.97		88.52	100.00
**Total**			0.25	19.30		80.46	100.00

For each of the 3 scores, the mean, standard deviation and the categories into which they were divided (≤ 2.5, between 2.5 and 3.5 and > 3.5) are provided

The 96.5% of physicians reported that RSV is a seasonal virus that, at our latitudes, causes epidemics, usually lasting 5 months, between November and March, with peaks in January/February. The 10.8% of paediatricians reported that RSV is transmitted only among children younger than 2 years of age. The 80.3% of participants thought that school-age children are not at risk of developing severe forms of RSV disease, 8% of participants thought that school-age children are at risk of developing severe forms of RSV disease, 11.7% were neutral. The 14.1% (*n* = 60) of paediatricians reported that apnoea is not the only presenting sign in infants diagnostic of RSV infection. The 68.9% of paediatricians reported that apnoea may be the only sign present in infants in the diagnosis of bronchiolitis, 17.1% is neutral and 14.1% think that apnoea is not the only sign. The 21.6% of physicians thought that most RSV hospitalizations occurred in children who have pre-existing pathological conditions (e.g., congenital heart disease and bronchopneumodysplasia), 17.1% were neutral and 61.4% reported that children hospitalised did not have pre-existing clinical conditions. The 73.8% of participants thought that most RSV hospitalizations occurred in healthy, preterm and full-term infants born not eligible for current prophylaxis, with rates between 70 and 90%. The 95.1% of physicians thought that all newborns and infants in their first RSV season are at risk of developing RSV infection such as bronchiolitis, during the period of virus circulation and 77.1% believed that newborns and infants would all need protection in their first RSV season because it is impossible to predict which children might make an RSV infection with need for medical care. The 91.6% of respondents thought that forms of RSV infection, such as bronchiolitis, have been recognized as a risk factor for the development of bronchospasm (wheezing) and asthma in school-age-children and 79.6% believed that prevention of severe RSV infection, such as bronchiolitis, could prevent the following risk of developing bronchospasm and asthma in childhood.

#### Knowledge of monoclonal antibodies

Average mAb knowledge score was 3.70 (SD = 0.45). The questions about mAb knowledge which obtained the lowest average score were **Q1** (At present, to prevent Respiratory Syncytial Virus, an active form of immunization– for all newborns and children aged < 2, i.e., vaccine, is available; mean = 3.69, DS = 1.28) and **Q2** (Currently, there is a form of passive immunization to protect all newborns and children under 2 years of age from the Respiratory Syncytial Virus; mean = 2.67, DS = 1.32). The question about mAb knowledge which obtained the highest average score was **Q4** (Current prophylaxis with Palivizumab has demonstrated significant efficacy against RSV in terms of reducing hospitalisations and outpatient medical care; mean = 4.22; SD = 0.61) [Additional file 1].

The 25% of participants (*n* = 101) answered that, at the current state, a form of active immunization, i.e., vaccine, is available to prevent RSV; 5.5% were neutral and 69.6% thought that a vaccine is not yet available. The 57.2% (*n* = 231) of paediatricians thought that, at present, there is a form of passive immunization for the protection of all infants and children under 2 years of age from RSV; 7.2% were neutral and 35.7% thought that a passive immunization is not yet available. The 76.5% thought that current prophylaxis with palivizumab can be used in all infants born preterm and in all those born with high-risk clinical conditions and 94.6% believed that current prophylaxis with palivizumab has demonstrated significant efficacy against RSV in terms of reduced hospitalizations and outpatient medical care. The 69.6% of physicians answered that the mAb Nirsevimab is designed to protect all infants and children in their first season of RSV; 25.5% were neutral and 5.2% thought is not designed for that population target. The 76.2% of participants thought that Nirsevimab has shown significant efficacy against RSV in terms of reducing hospitalizations and outpatient medical care. The 22.3% of respondents were neutral and 1.5% thought that Nirsevimab has not show that efficacy.

### Position towards immunization strategies (attitudes)

Average mAb attitude score was 4.20 (SD = 0.62). The question about mAb attitude which obtained the lowest average score were **Q2** (I would support the use of a monoclonal antibody in all infants and children in their first season of RSV if it is available, safe, and cost-effective; mean = 4.09, DS = 0.82). The question about attitude towards mAb score which obtained the highest average score were **Q1** (I would support the use of a vaccine against RSV in all children if it is available, safe, and cost-effective; mean = 4.26; DS = 0.74) and **Q3** (I would be in favour of an RSV prevention strategy in all newborns and children because it is also useful for preventing RSV complications during childhood, such as bronchospasm and asthma; mean = 4.26; DS = 0.66) [Additional file 1].

The 92.1% (*n* = 361) of physicians would support the use of a vaccine against RSV if it were available, safe, and cost-effective, 3.6% were neutral and 4.5% disagreed with this statement whilst 82.4% would support the use of monoclonals against RSV. The 82.4% of respondents would support the use of mAbs in all infants and children in their first season of RSV if available, safe, and cost-effective. The 11.7% of physicians were neutral and none of them disagreed with this statement. The 92.1% of paediatricians were in favour of a prevention strategy against RSV because useful in preventing complications of the virus during childhood such as bronchospasm and asthma.

### Behaviours toward treatment and prevention of RSV infection (practises)

The 97.5% of physicians managed bronchiolitis cases. Of these, 53.1% saw more than 10 cases in the past season. The 93.8% of family paediatric practitioners had hospitalized patients. The 65.6% of these physicians did not request the administration of monoclonal antibodies and 65% reported it was possible to immunize infants with monoclonals before the start of the epidemic season (between April-October). The 90% of hospital physicians saw an overall advance and increase in the number of hospitalized children in the 21–22 season. In 90% of cases, hospital physicians mentioned RSV as the main cause of hospitalization for respiratory infections between November and March in infants < 1 year old. In 37.7% of cases, infants born healthy, preterm or term who were not eligible for current prophylaxis accounted for 80–90% of those hospitalized. The 78.2% of hospital physicians believed it was possible to immunize infants (born between November and March) with monoclonals upon discharge from the birth point.

### Inferential analysis

To the highest category of RSV knowledge score (> 3.5) belonged mostly women compared to men (90.9% vs. 88.6%; *p* = 0.47), hospital-based paediatricians (91.3% vs. 88.9%, *p* = 0.43), paediatricians aged 30–45 years (93.9%, *p* = 0.06) and specialized from > 5 years (93.2%, *p* = 0.89), coming from Centre (91.8%), having handled 0 or 5–10 cases in the previous season (92.3%, *p* = 0.64). However, none of these associations was statistically significant.

In the highest RSV knowledge score category (> 3.5), the least represented were the paediatricians aged < 30 years (78.6%, *p* = 0.06), specialized from 11 to 20 years (87.7%, *p* = 0.89), coming from Islands (81.3%, *p* = 0.52), who handled 15–20 cases the previous season (77.8%, *p* = 0.64). However, none of these associations was statistically significant. Paediatricians with higher mAb knowledge scores belonged mostly to the higher category of RSV knowledge score (93.6%, *p* < 0.001).

To the highest category of mAb knowledge score (> 3.5) belonged mostly men compared to women (67.3% vs. 60.34%, *p* = 0.38), the paediatricians aged 56–65 years (72.7%, *p* < 0.001) and specialized from > 20 years (70.7%, *p* = 0.001), coming from South (75%, *p* = 0.15), who handled < 5 cases in the previous season (73.9%, *p* = 0.07), hospital-based (63% vs. 61.11%, *p* = 0.64). However, there was statistical significance only for age and professional seniority.

In the highest category of mAb knowledge score (> 3.5), the least represented were the Paediatrics residents (51.7%, *p* < 0.001), the paediatricians aged 46–55 years (53%, *p* < 0.001), coming from North-West (54.6%, *p* = 0.15), who handled 15–20 cases the previous season (33.3%, *p* = 0.07). However, there was statistical significance only for being a resident and age. Paediatricians with higher RSV knowledge scores belonged mostly to the highest category of mAb knowledge score (64.7%, *p* < 0.001).

The logistic regression model found that, holding all other predictor variables constant, the odds of a positive attitude towards mAB knowledge score increased by 223% (OR 3.229, 95% CI [1.41, 7.395], *p* = 0.006) for being female. Similarly, holding all other predictor variables constant, the odds of a positive attitude towards mAB knowledge score increased by 873% (OR 9.725, 95% CI [3.062, 30.892], *p* < 0.001) for a one-unit increase in RSV knowledge score. Finally, holding all other predictor variables constant, the odds of a positive attitude towards mAb knowledge score increased by 291% (OR 3.91, 95% CI [1.538, 9.939], *p* = 0.004) for a one-unit increase in mAb knowledge score. **[**Table 3]

**Table 3:** Logistic regression

Logistic regression							
Mean attitude towards monoclonal antibody use	Coefficient	Standard Error	t-value	*p*-value	[95% Confidence	Interval]	Significance
**Mean knowledge about RSV**	9.725	5.735	3.86	0	3.062	30.892	***
**Mean knowledge about monoclonal antibody passive immunization**	3.91	1.861	2.86	0.004	1.538	9.939	***
**Gender**							
Male	1	.	.	.	.	.	
Female	3.229	1.365	2.77	0.006	1.41	7.395	***
**Age**							
< 30	1	.	.	.	.	.	
30–45	0.302	0.385	-0.94	0.347	0.025	3.661	
46–55	0.209	0.272	-1.20	0.23	0.016	2.692	
56–65	0.242	0.318	-1.08	0.28	0.018	3.17	
> 65	0.591	0.805	-0.39	0.699	0.041	8.54	
**Work setting**							
Hospital	1	.	.	.	.	.	
Community	1.493	0.612	0.98	0.328	0.669	3.335	
**Geographical region**							
North-West	1	.	.	.	.	.	
North-East	1.251	0.574	0.49	0.625	0.509	3.076	
Center	1.849	0.959	1.18	0.236	0.669	5.112	
South	2.932	2.055	1.53	0.125	0.742	11.585	
Islands	1.23	0.777	0.33	0.743	0.357	4.24	
**Constant**	0	0	-4.31	0	0	0.002	***
Mean dependent variable		0.887		SD dependent variable		0.317	
Pseudo r-squared		0.191		Number of observations		388	
Chi-square		52.439		Probability > chi2		0.000	
Akaike criterion (AIC)		247.931		Bayesian criterion (BIC)		299.424	

*** *p* < 0.01, ** *p* < 0.05, * *p* < 0.1

The Table 3 shows results obtained from logistic regression. Gender, age, job setting, geographical region, mean RSV knowledge score and mean mAb knowledge score were included in the logistic regression as independent variables.

## Discussion

The present study aimed at assessing Italian paediatricians’ knowledge, attitudes and behaviours towards RSV and its preventive strategies. The results obtained confirm heterogeneity of perspectives among the selected sample. The gathered data show substantial comparability to findings in earlier studies, even upon direct comparison.

Indeed,the findings of this study align with those of two other recent similar Italian works [32–33]. The first highlighted unsatisfactory knowledge among general practitioners regarding RSV epidemiology and prevention with mAb. The second one found lack of personal expertise of the sampled professionals, not only on mAb, but also regarding RSV cases.

All the three studies are lined up regarding the high level of acceptance of a future RSV vaccine and knowledge about the virus’s epidemic periods.

In our study most of respondents were female and got specialization for more than 20 years. During their clinical practice, most of them managed numerous RSV cases in the past year, and many of them have confirmed a recurrence of the same cases in the period 2021/2022, similarly to Riccò et al. 2023 [34]. The study may support the hypothesis of immunological debt due to the recent SARS-CoV-2 pandemic although further investigations are needed to prove it. Some paediatricians thought school-age children were not at risk of severe RSV infection. Moreover, they struggled to identify diagnostic signs like apnoea and risk factors leading to hospitalization. In our study, 60 out of 427 (14%) participants disagreed that apnoea is the only diagnostic sign of bronchiolitis.

Our results showed that there is a lack of knowledge on RSV, which is likely to affect the use of preventive strategies. The lack of knowledge is also evidenced by the fact that some physicians did not know how to define what specific target the Nirsevimab antibody is aimed at and whether it demonstrated efficacy in terms of reducing hospitalizations and local health care. About clinical practice, although more than 65% of paediatricians did not request monoclonal antibodies for their patients (vs. 5,1% [33] 2021 vs. 14.4% in [34] 2022), 65% of the participants believed it is possible to administer them in the future at recall visits for health checkups before the start of the epidemic season. These positions may indicate fear and reluctance regarding the use of new preventive strategies although scientific evidence suggests the opposite. Considering these findings, an information campaign aimed at physicians regarding possible options to employ against RSV appears even more advised.

Our results showed that older age and seniority for paediatricians were associated with better knowledge.

In the same category previously considered the least represented were the Paediatrics residents and the paediatricians aged 46–55 years. There was statistical significance only for being a resident and age. Paediatricians with higher RSV knowledge scores belonged mostly to the highest category of mAb knowledge score. This suggests that paediatricians with higher RSV knowledge are also more informed about the use of mAb as valid preventive strategies and maybe they are more in favour to introduce mAb in their practice.

Furthermore, considering the logistic regression, results regarding attitudes and their connection with RSV and mAb knowledge score found that the probability of having a positive attitude about mAb knowledge score is significantly higher if paediatricians are female and increases exponentially with an increase in RSV knowledge score. This is an interesting hint since being female and acquiring RSV expertise could lead to an increasing use of mAb as preventive strategy.

With reference to our Italian national scenario, the existence of gaps in knowledge, attitudes and inappropriate behaviours adopted by some paediatricians could play a negative role on Public Health measures. Poor embracement of these preventive measures could lead to severe cases of RSV infection and to indirect health spending cost growth due to healthcare services. Italian National Health Service ensures the Universal Health Coverage and for this reason it is important not to burden it in order to guarantee accessibility of health coverage to the whole population.

It would be interesting to expand our research with a European or international outlook to assess whether substantial divergences or alignments in results are present. Examining for instance the Spanish situation, mAbs were recently introduced in 2023 in the Paediatric Immunization Calendar [35]. This choice demonstrates how the Spanish view may be different from the Italian one, and we can undoubtedly not exclude the possibility that other countries take different positions that would therefore deserve to be studied in depth. It would also be interesting to conduct an in-depth study focusing on some variables, such as female gender, which obtained a positive association with knowledge of mAb and RSV, or the additional specializations of paediatricians, to test a possible relationship between paediatricians having specialization in infectious diseases or public health and awareness and knowledge regarding RSV and its preventive strategies.

Although there are several preventive strategies available for RSV prevention, the limited knowledge, the doubtful behaviours, and negative attitudes of paediatricians may be an obstacle to its implementation. In order to increase awareness in the population, an educational program aimed at both paediatricians and public health professionals could be a useful option. In addition, it would also be appropriate to involve Prevention Departments in dissemination since increasing patient adherence to preventive strategies, to improve well-being and health in the community, is public health physician task. Cooperation between paediatricians and public territory health physicians should be built with a view to reach this goal. Public health physicians can offer their expertise in RSV immunization and prevention to paediatricians through various ways. They could provide evidence-based guidelines and information, build a collaborative research, set up workshops and conferences and they could create resource development (e.g., educational materials) addressed to paediatricians and their patients. Finally, as it is crucial to impact less on health care budget and thus on the National Health Service since acquisition of RSV infection costs are not insignificant quantifying in 4.8 billion euros [22]. The strengths of the study are several: the considerable sample size, which returns us a strong statistical representativeness, the geographical representativeness and the originality and wideness. Although is not the first work on RSV prevention in Italy, the topical slant and the variety of questions, the way participants are recruited in the questionnaire make our study different from the previous ones.

However, some limitations should be considered as well. First, the survey results could be influenced by the “self-selection” of the participants. In this way, the data obtained could be polarized and may not be generalizable in terms of attitudes, knowledge and behaviours to the whole population defined as the whole number of specialists in Paediatrics working in Italy. Regarding the sample size, only 507 paediatricians completed the questionnaire, roughly accounting for 4.5% of all SIP paediatricians. It is important to note that the results derived from this survey cannot be broadly applied to all paediatricians participating in the study. Hence, further research is necessary. However, it is customary in studies like this to confront the challenge of statistical significance by selecting a smaller sample that aims to represent the entire population. Additionally, our study may be subject to sampling selection bias since respondents are more likely to be attentive to these topics and more responsive to SIP calls. Lastly, we must clarify that the following study was conducted on a national, and not a European or international basis. Finally, being survey-oriented, there are structural methodological limitations compared to other epidemiological analytical studies. Finally, it should be noticed that a wider sample including also neonatologists, midwives and obstetricians would have been more useful to obtain generalizable results, considering that the new monoclonal antibody for RSV immunization is supposed to be primarily administered at birth or during the first months of life [22–23, 28] and the target for the RSV vaccine to protect infants against RSV in the first six months of life and just approved in Spain is currently represented by pregnant women [24–25].

## Conclusions

Respiratory Syncytial Virus primarily affects newborns, infants and young children under the age of two years. The current prevention method involves passive immunization using monoclonal antibodies. The findings from the present study indicate a lack of understanding about RSV, potentially hindering the use of preventive strategies. Knowledge gaps, inappropriate behaviours, and attitudes among some paediatricians could negatively impact Public Health measures. Collaboration between public health physicians and paediatricians is suggested to improve RSV prevention through educational programs, and reducing RSV infection costs is crucial for the sustainability of the Italian National Health Service.

### Electronic supplementary material

Below is the link to the electronic supplementary material.

**Table S1**: Likert scale values

## Data Availability

The datasets used and/or analysed during the current study are available from the corresponding author on reasonable request.

## Abbreviations

RSV: Respiratory Syncytial Virus
mAb: Monoclonal antibodies
LTRI: Lower respiratory tract infection
ICU: Intensive Care Unit
CHD: Congenital Heart Disease
CLD: Chronic Lung Disease
CHMP: Committee for Medicinal Products for Human Use
EMA: European Medicine Agency
FDA: Food and Drug Administration
AIFA: Agenzia Italiana del Farmaco
ISTAT: Italian National Institute of Statistics
SIN: Italian Society of Neonatology
SIP: Italian Society of Pediatrics
SIMRI: Italian Society for Infant Respiratory Disease
NHS: National Health System
