# Supplementary material for: Knowledge, attitudes and behaviours of a sample of Italian paediatricians towards RSV and its preventive strategies: a cross-sectional study
Source: Ital J Pediatr. 2024 Feb 29;50:35. doi: 10.1186/s13052-024-01593-1 (PMC10905893; doi:10.1186/s13052-024-01593-1)
Supplement: Supplementary file 1 — Table S1: Likert scale values [file 13052_2024_1593_MOESM1_ESM.docx]

**Table S1. Likert scale values**

| **Variable** | | **Values** | | | | | |
| --- | --- | --- | --- | --- | --- | --- | --- |
|  |  | 1 | 2 | 3 | 4 | 5 | **Total** |
|  | **RSV Knowledge** |  | | | | | |
| **Q1** | The Respiratory Syncytial Virus is a seasonal virus that, in our latitudes, causes epidemics, usually lasting 5 months, between November and March, with peaks in January/February. | 1.17 | 0.70 | 1.64 | 48.71 | 47.78 | 100.00 |
| **Q2** | The Respiratory Syncytial Virus is transmitted only among children under 2 years of age. | 3.04 | 7.73 | 4.92 | 49.88 | 34.43 | 100.00 |
| **Q3** | School-age children are at risk of developing severe forms of RSV disease. | 0.47 | 7.49 | 11.71 | 56.21 | 24.12 | 100.00 |
| **Q4** | In children under 1 year of age, initial symptoms may include irritability, reduced activity, and apnoea. | 0.47 | 4.92 | 7.49 | 57.14 | 29.98 | 100.00 |
| **Q5** | Inability to feed is a criterion for hospital admission for a child affected by RSV. | 0.70 | 1.17 | 1.17 | 50.82 | 46.14 | 100.00 |
| **Q6** | In the diagnosis of bronchiolitis, apnoea may be the only sign present in infants. | 1.17 | 12.88 | 17.10 | 51.05 | 17.80 | 100.00 |
| **Q7** | The majority of hospitalisations for RSV occur in children who have pre-existing pathological conditions, such as congenital heart disease and bronchopulmonary dysplasia. | 5.39 | 16.16 | 17.10 | 49.41 | 11.94 | 100.00 |
| **Q8** | The majority of hospitalisations for RSV occur in infants born healthy, both preterm and full-term, who are not eligible for current prophylaxis, with rates between 70% and 90%. | 1.17 | 11.24 | 13.82 | 59.72 | 14.05 | 100.00 |
| **Q9** | All newborns and infants experiencing their first RSV season are at risk of developing RSV infections, such as bronchiolitis, during the period of virus circulation. | 0.00 | 1.87 | 3.04 | 63.00 | 32.08 | 100.00 |
| **Q10** | Newborns and infants in their first RSV season would all require protection because it is impossible to predict which children might become infected with RSV and require medical assistance. | 0.47 | 9.37 | 13.11 | 55.74 | 21.31 | 100.00 |
| **Q11** | Forms of RSV infection, such as bronchiolitis, have been recognised as a risk factor for the development of bronchospasm (wheezing) and asthma in school-age children. | 0.47 | 2.34 | 5.62 | 61.12 | 30.44 | 100.00 |
| **Q12** | Preventing severe RSV infection, such as bronchiolitis, could prevent the subsequent risk of developing bronchospasm and asthma during childhood. | 0.47 | 5.39 | 14.52 | 61.59 | 18.03 | 100.00 |
|  | **mAb Knowledge** |  | | | | | |
| **Q1** | Currently, there is an active form of immunisation, namely a vaccine, available to prevent the Respiratory Syncytial Virus. | 7.18 | 17.82 | 5.45 | 37.87 | 31.68 | 100.00 |
| **Q2** | Currently, there is a form of passive immunisation to protect all newborns and children under 2 years of age from the Respiratory Syncytial Virus. | 21.04 | 36.14 | 7.18 | 26.24 | 9.41 | 100.00 |
| **Q3** | Current prophylaxis with Palivizumab can be used in all children born preterm and those born with high-risk clinical conditions. | 1.98 | 14.60 | 6.93 | 48.02 | 28.47 | 100.00 |
| **Q4** | Current prophylaxis with Palivizumab has demonstrated significant efficacy against RSV in terms of reducing hospitalisations and outpatient medical care. | 0.50 | 0.99 | 3.96 | 64.85 | 29.70 | 100.00 |
| **Q5** | The monoclonal antibody Nirsevimab has been studied for the protection of all newborns and children in their first RSV season. | 0.74 | 4.46 | 25.25 | 50.00 | 19.55 | 100.00 |
| **Q6** | The monoclonal antibody Nirsevimab has demonstrated significant efficacy against RSV in terms of reducing hospitalisations and outpatient medical care. | 0.25 | 1.24 | 22.28 | 56.93 | 19.31 | 100.00 |
|  | **Attidudes** |  |  |  |  |  |  |
| **Q1** | I would support the use of a vaccine against RSV in all children if it is available, safe, and cost-effective. | 0.51 | 3.83 | 3.57 | 53.57 | 38.52 | 100.00 |
| **Q2** | I would support the use of a monoclonal antibody in all newborns and children in their first RSV season if it is available, safe, and cost-effective. | 0.00 | 5.87 | 11.73 | 49.74 | 32.65 | 100.00 |
| **Q3** | I would be in favour of an RSV prevention strategy in all newborns and children because it is also useful for preventing RSV complications during childhood, such as bronchospasm and asthma. | 0.00 | 2.04 | 5.87 | 56.38 | 35.71 | 100.00 |
| **Total** | | 2.22 | 7.99 | 9.69 | 52.84 | 27.26 | 100.00 |

Table legend: Q = Question

The table shows questions belonging to 3 categories. It is a quantitative projection of the data coming from Likert-scale questions reported according to RSV knowledge, mAb knowledge and mAb attitude scores for each participant, by calculating mean and SD of the Likert-type statements related to each dominion.
